# Enhancing explainable SARS-CoV-2 vaccine development leveraging bee colony optimised Bi-LSTM, Bi-GRU models and bioinformatic analysis

**DOI:** 10.1038/s41598-024-55762-7

**Published:** 2024-03-20

**Authors:** Dilber Uzun Ozsahin, Zubaida Said Ameen, Abdurrahman Shuaibu Hassan, Auwalu Saleh Mubarak

**Affiliations:** 1 Department of Medical Diagnostic Imaging, College of Health Science, University of Sharjah, Sharjah, UAE; 2 Research Institute for Medical and Health Sciences, University of Sharjah, Sharjah, UAE; 3 Operational Research Centre in Healthcare, Near East University, TRNC Mersin 10, Nicosia, 99138 Turkey; 4 Department of Electrical Electronics and Automation Systems Engineering, Kampala International University, Kampala, Uganda; 5 Department of Biochemistry, Yusuf Maitama Sule University, Kano, Nigeria; 6 Department of Electrical Engineering, Aliko Dangote University of Science and Technology, Wudil, Kano, Nigeria

**Keywords:** Computational biology and bioinformatics, Drug discovery, Immunology

## Abstract

The severe acute respiratory syndrome coronavirus 2 (SARS-CoV-2) is a single-stranded RNA virus that caused the outbreak of the coronavirus disease 2019 (COVID-19). The COVID-19 outbreak has led to millions of deaths and economic losses globally. Vaccination is the most practical solution, but finding epitopes (antigenic peptide regions) in the SARS-CoV-2 proteome is challenging, costly, and time-consuming. Here, we proposed a deep learning method based on standalone Recurrent Neural networks to predict epitopes from SARS-CoV-2 proteins easily. We optimised the standalone Bidirectional Long Short-Term Memory (Bi-LSTM) and Bidirectional Gated Recurrent Unit (Bi-GRU) with a bioinspired optimisation algorithm, namely, Bee Colony Optimization (BCO). The study shows that LSTM-based models, particularly BCO-Bi-LSTM, outperform all other models and achieve an accuracy of 0.92 and AUC of 0.944. To overcome the challenge of understanding the model predictions, explainable AI using the Shapely Additive Explanations (SHAP) method was employed to explain how Blackbox models make decisions. Finally, the predicted epitopes led to the development of a multi-epitope vaccine. The multi-epitope vaccine effectiveness evaluation is based on vaccine toxicity, allergic response risk, and antigenic and biochemical characteristics using bioinformatic tools. The developed multi-epitope vaccine is non-toxic and highly antigenic. Codon adaptation, cloning, gel electrophoresis assess genomic sequence, protein composition, expression and purification while docking and IMMSIM servers simulate interactions and immunological response, respectively. These investigations provide a conceptual framework for developing a SARS-CoV-2 vaccine.

## Introduction

A worldwide health emergency brought on by the coronavirus disease 2019 (COVID-19) pandemic has driven research to create effective vaccines against severe acute respiratory syndrome coronavirus 2 (SARS-CoV-2), the etiological agent of COVID-19^1,2^. The World Health Organisation (WHO) declared COVID-19 a pandemic on March 11, 2020, due to its global spread^3^. Four structural proteins make up the virus: spike (S), envelope (E), nucleocapsid (N), and membrane (M). These structural proteins are crucial for the virus’s entrance into host cells and the subsequent segregation of its particles^4^. Immune responses, particularly creating antibodies against the structural proteins, are crucial to preventing infection. An efficient and safe vaccine is the safest and most regulated approach to prevent COVID-19. Developing a vaccine and quickly scaling it up for mass manufacturing during a global pandemic is challenging. Thus, it is imperative to accelerate vaccine development using new technological platforms^5^. For vaccine design, four approaches continue to be the top choices: (1) Nucleic acid vaccines are s generated when the viral genome is sequenced. (2) Proteins based on viral vectors. (3) Viruses destroyed by heat or UV radiation or tumorous components are used in conventional inactivated viral vaccines, and (4) Recombinant peptide subunit vaccines use viral antigenic epitopes. However, The first three are not recommended for immunocompromised individuals, who comprise most of the COVID-affected population. Therefore, COVID-19 vaccine development has drawn attention to peptide-based vaccines, which employ unique antigenic areas called epitopes to elicit an immune response. Since epitope-based are less allergenic and have lower production costs than other methods, epitope vaccines have become increasingly popular^6^. An epitope is an antigen molecule recognised by antibodies or T and B cells in the human immune system. Epitope recognition is crucial in epitope-based vaccine design to control pandemics brought on by the spread of infectious diseases like COVID-19^7,8^. Epitopes are essential for producing antibodies and boosting the human immune system. There are two epitopes: B-cell epitopes recognised by B-cells and T-cell epitopes presented to CD8 and CD4 T cells. An antigen is directed towards the CD4 T cells by MHC-II upon entry into the cells. Also, antigens are presented to the cytotoxic CD8 T cells through MHC-I produced by antigen-presenting cells (APCs) to kill infected cells. On the other hand, B-cells produce antibodies when stimulated by the CD4 T cells^9^. Attempts have been made to design and manufacture peptide vaccines that target immunogenic epitopes on the viral structural proteins To assist immune cells in recognising these crucial viral epitopes quickly^10–12^. Thus, in order to create vaccines against the virus, it is crucial to select the SARS-CoV-2 viral epitopes that will stimulate effective T-helper (HTL), T-cytotoxic (CTL), and B-cell activation^8^.

Despite this, finding highly antigenic epitopes involves experimental testing and is challenging, expensive, and time-consuming. A popular and successful option in this area is the use of computational tools to predict epitopes and assess the characteristics they possess. Many computational methods have been developed to predict epitopes from protein sequences, such as structural, sequence-based, and machine learning-based techniques. Bioinformatics and immune-informatics have grown in response to this pressing requirement to study and characterise proteins, creating a more substantial potential for vaccine development^13–15^. Several immuno-informatics techniques were integrated to generate a list of potentially immunogenic and antigenic peptide epitopes that might help develop vaccines^16–23^. A multi-epitope vaccine was developed by^24^ using the immunoinformatic method to predict several proteins from the SARS-CoV-2 proteome likely to cause an immune response. It was possible to determine which antigenic areas of the SARS-CoV-2 S protein from B.1.1.529 could induce B-cell and T-cell immunity.

Additionally, Bhatt et al.^25^ created a computational pipeline that predicts T cell epitopes in SARS-CoV-2 proteins by combining sequence-based and structure-based methods. In another study, vaccine designs featuring T- and B-cell epitopes were selected after examining the spike proteins’ S1 and S2 areas^26^. Utilising immunoinformatic strategies, it was possible to identify putative epitopes from SARS-CoV-2 that can generate immune responses critical to creating COVID-19 vaccines. Fifteen putative immune-stimulating areas and 25 epitopes 100% similar to SARS-CoV epitopes confirmed by the experiment were identified. Analysis was done to see if the epitopes would work as a vaccine^27^. This is similar to the immunoinformatic strategy used to generate a multi-epitope COVID-19 vaccine that can be used for both defensive and preventive measures. The multi-epitope vaccine was created by integrating the HTL, CTL, and B cell epitopes. An additional study was done to use internet resources to predict and evaluate the composition and efficacy of the vaccine^28^.

Immunoinformatic approaches for vaccine creation have drawbacks since they automatically compute thousands of regions for epitope selection when just a few are required. For epitope prediction, machine learning (ML) techniques have the potential to distinguish between different epitopes quickly based on a variety of features^29^. As a result, there are machine learning (ML) studies and techniques that use the features to predict epitopes using ensemble ML^30^ and decision tree-based ensemble^31^. Furthermore, Nisar et al.^30^ suggested a computational approach to create T-cell peptide-based vaccines against SARS-CoV-2 using ensemble machine learning-based approaches. They also discovered several prospective peptide vaccines that might be further verified using experimental tests. The use of deep learning (DL) approaches as screening tools for COVID-19 identification has shown great promise. DL efficiently reduces time, expense, and burden on COVID-19 diagnosis^32–34^. For instance, Yang et al.^35^, Ameen et al.^36^, Abbasi et al.^37^ suggested computational and deep learning approaches to create multi-epitope-based vaccines against SARS-CoV-2. They found several potential peptide vaccines that may be further tested utilising experimental studies, in addition to showing that their technique creates vaccine candidates with useful immunogenicity and minimal toxicity. Despite being regarded as successful, these models’ performances need to be improved.

Furthermore, the evaluation of the overlapping fragments’ biological characteristics is laborious. To choose the best viral protein epitopes for creating a successful vaccine, we must wholly and carefully evaluate all the predicted data^38,39^, which adds significant overhead and can take much time. Deep learning methods presented in this work will provide a fast and efficient tool for predicting epitopes for designing multi-epitope vaccines.

For the classification of peptides into epitopes or non-epitopes, this study proposes a hybrid deep learning model based on Convolutional Neural Networks (CNN), Bidirectional Long Short-Term Memory (Bi-LSTM), Bidirectional Gated Recurrent Unit (Bi-GRU) and bioinspired optimisation algorithms Bee Colony Optimization (BCO). Compared to other machine learning models trained on the same datasets, the model performs well and shows good accuracy. Furthermore, we successfully express the vaccine in *Escherichia coli* using in-silico cloning and codon optimisation and analyse the anticipated epitopes’ toxicity, antigenicity, and allergenicity using bioinformatics methods. This research offers a viable method for identifying and assessing possible COVID-19 vaccine epitopes. The deep learning model’s lack of interpretability is one of the primary worries, especially in vaccine design. Thus, the necessity for making them more interpretable is growing, especially in this area. First, it is crucial to ensure that model predictions are based on trustworthy representations. Therefore, for vaccine design, it is necessary to understand and trust the neural network’s judgment, which is only possible with the interpretability requirement being satisfied. Otherwise, the lives of humans can be in danger. In this work, we identified the essential features for predicting epitopes and the role of all features using the Shapely Additive Explanations (SHAP) method.

Here are some novel research contributions for this study on epitope prediction for SARS-CoV-2 vaccines:

1. In the first stage of the study, Recurrent Neural Networks (Bidirectional Long Short-Term Memory (Bi-LSTM) and Bidirectional Gated Recurrent Unit (Bi-GRU)) were built from scratch.
2. Novel hybrid models (CNN-Bi-LSTM, CNN-Bi-GRU, BCO-Bi-LSTM, BCO-Bi-GRU, BCO-CNN-Bi-LSTM, BCO-CNN-Bi-GRU) were developed to improve the performance of the models and find the best-performing model.
3. To increase the accuracy of epitope prediction, numerous additional information was used besides the SARS-CoV-2 proteins’ sequence information. For instance, we integrated structural and chemical information to offer a more thorough picture of probable epitopes and boost prediction accuracy.
4. To demonstrate the quality of the vaccine, we further examine its toxicity, potential antigenic properties, possibility of allergic reactions, and other biochemical parameters.
5. An adaptation of codons and cloning are also employed to examine the vaccine’s genomic sequence and protein composition and guarantee its efficient expression. After the vaccine’s 3D structure was generated with I-TASSER and galaxyWeb, docking was used to show how the vaccine interacts with its receptor. Finally, using the IMMSIM server, the immunological response anticipated from the vaccination was simulated.
6. An explainable AI technique was employed to comprehend how the unique method’s epitope predictions were created. Although deep learning methods have shown great promise in medical applications, they might be thought of as “black box” models because they do not reveal the process by which they make their predictions. Here, the SHAP technique was used to help researchers determine what attributes are most important for epitope classification. Explainable AI techniques have the potential to produce predictions that are simple to understand.

## Methods

### Datasets

The datasets used in this study were made publicly available and came from the Kaggle database. The SARS-CoV, B-cell, and SARS-CoV-2 datasets are all in the database (https://www.kaggle.com/datasets/futurecorporation/epitope-prediction). They have ten characteristics, with structural and chemical aspects comprising the data. Chou-Fasman (beta turn), Kolaskar-Tongaonkar (antigenicity), Parker (hydrophobicity), Emini (relative surface accessibility), Stability, isoelectric_point, Aromaticity, and Hydrophobicity are numerical. On the other hand, each protein sequence or peptide sequence will have a value corresponding to the number of their categorical letters. The sample of datasets can be visualised in Table 1.

**Table 1:** Sample of the datasets.

parent_protein_id	Protein-seq	start_position	end_position	peptide_seq	chou_fasman	……	emini	kolaskar_tongaonkar
A2T3T0	MDVLYSLSKTLKDAR	161	165	SASFT	1.016	……	0.703	1.018
F0V2I4	MTIHKVAINGFGRIGR	251	255	LCLKI	0.77	……	0.179	1.199
O75508	MVATCLQVVGFVTSF	145	149	AHRET	0.852	……	3.427	0.96
O84462	MTNSISGYQPTVTTST	152	156	SNYDD	1.41	……	2.548	0.936
P00918	MSHHWGYGKHNGPE	85	89	DGTYR	1.214	……	1.908	0.937
P00918	MSHHWGYGKHNGPE	155	159	GLQKV	0.928	……	0.547	1.09
P00918	MSHHWGYGKHNGPE	22	26	IAKGE	0.888	……	0.633	0.974

### Models

#### Bidirectional long short-term memory (Bi-LSTM)

Recurrent neural networks of the type known as Bidirectional Long Short-Term Memory (Bi-LSTM)^40^ can handle sequential data in both forward and backward orientations. The Bi-LSTM is primarily employed in studies involving speech recognition and natural language processing, where understanding each word’s context is crucial. The Bi-LSTM model consists of two LSTM layers, one of which processes the input sequence forward and the other of which processes it backwards. The final output is created by concatenating the results of each layer.

The forward and reverse states are calculated by the Bi-LSTM model using the following equations:

a. Forward LSTM equations:

1 $$I_{t} = sigma(W_{xi} x_{t} + W_{hi} h_{t} - 1 + b_{i}$$

2 $$f_{t} = sigmaW_{xf} x_{t} + w_{f} h_{t - 1} + b_{f}$$

3 $$c_{t} = f_{t} *c_{t - 1} + i_{t} *\tanh \left( {W_{xc} x_{t} + W_{hc} h_{t - 1} + b_{c} } \right)$$

4 $$o_{t} = sigma(W_{x0} x_{t} + W_{h0} h_{t - 1} + b_{0}$$

5 $$h_{t} = o_{t} *\tanh \left( {c_{t} } \right)$$

where *i*_*t*_, *f*_*t*_, and o_t_ are the input, forget, and output gates, and *W* and *b* are the weights and biases of the LSTM layer. *x*_*t*_ is the input sequence at time *t. h*_*t*_ and *c*_*t*_ are the hidden state and cell state at time *t.*

b. Backward LSTM equations:

6 $$i^{\prime}_{t} = sigma\left( {W^{\prime}_{hi} h^{\prime}_{t + 1} + b^{\prime}_{i} } \right)$$

7 $$f^{\prime}_{t} = sigmaW^{\prime}_{xf} x^{\prime}_{t} + w^{\prime}_{f} h^{\prime}_{t - 1} + b^{\prime}_{f}$$

8 $$c^{\prime}_{t} = f^{\prime}_{t} *c^{\prime}_{t - 1} + i^{\prime}_{t} *\tanh\!\!\left( {W^{\prime}_{xc} x^{\prime}_{t} + W^{\prime}_{hc} h^{\prime}_{t - 1} + b^{\prime}_{c} } \right)$$

9 $$o^{\prime}_{t} = sigma(W^{\prime}_{x0} x^{\prime}_{t} + W^{\prime}_{h0} h^{\prime}_{t - 1} + b^{\prime}_{0}$$

10 $$h^{\prime}_{t} = o^{\prime}_{t} *\tanh \left( {c^{\prime}_{t} } \right)$$

where *x'*_*t*_ is the input sequence at time *t* in the backward direction, *h'*_*t*_ and *c'*_*t*_ are the hidden state and cell state at time *t* in the backward direction, *i'*_*t*_*, f'*_*t*_, and *o'*_*t*_ are the input, forget, and output gates, and *W'* and *b'* are the weights and biases of the backward LSTM layer.

The forward and backward hidden states at each time step are concatenated to create the Bi-LSTM model’s final output, which is then processed through a fully connected layer to provide the final prediction.

In conclusion, the Bi-LSTM model is a kind of recurrent neural network applied to sequential input’s forward and backward processing. The model’s two LSTM layers compute the forward and backward states. Based on the input sequence and the model’s weights and biases, the equations employed in the Bi-LSTM model update the hidden and cell states of the LSTM layers. The forward and backward hidden states of the Bi-LSTM model are combined, and then they are sent through a fully connected layer to get the final output.

#### The bidirectional gated recurrent unit (Bi-GRU)

The Bidirectional Gated Recurrent Unit (Bi-GRU)^41^ is a type of recurrent neural network (RNN) that can capture dependencies in both the forward and backward directions. It is commonly used in tasks that involve sequential data analysis, such as natural language processing and speech recognition. The Bi-GRU model consists of two GRU layers: one that processes the input sequence in the forward direction and another in the backward direction. The outputs of both layers are combined to form the final output of the Bi-GRU model.

11 $$r_{t} = \sigma (W_{r} *x_{t} + U_{r} *h_{t - 1} + b_{z}$$

12 $$z_{t} = \sigma (W_{z} *x_{t} + U_{z} *h_{t - 1} + b_{z}$$

13 $$h_{t} = tanh(W_{w} *x_{t} + r_{t} \odot \left( {U_{h} *h_{t - 1} } \right) + b_{h}$$

14 $$h_{t} = \left( {1 - z_{t} \odot h_{t - 1} + z_{t} \odot h_{t} } \right)$$

where Reset Gate *is r*_*t*_, Update Gate *z*_*t*_, Candidate Activation h_t_, Hidden State *h*_*t*_.

Similarly, the backward GRU layer has its own set of equations:

15 $$r^{\prime}_{t} = \sigma (W^{\prime}_{r} *x^{\prime}_{t} + U^{\prime}_{r} *h^{\prime}_{t - 1} + b^{\prime}_{z}$$

16 $$z^{\prime}_{t} = \sigma (W^{\prime}_{z} *x^{\prime}_{t} + U^{\prime}_{z} *h^{\prime}_{t - 1} + b^{\prime}_{z}$$

17 $$h^{\prime}_{t} = tanh(W^{\prime}_{w} *x^{\prime}_{t} + r^{\prime}_{t} \odot \left( {U^{\prime}_{h} *h^{\prime}_{t - 1} } \right) + b^{\prime}_{h}$$

18 $$h^{\prime}_{t} = \left( {1 - z^{\prime}_{t} \odot h^{\prime}_{t - 1} + z^{\prime}_{t} \odot h^{\prime}_{t} } \right)$$

where Reset Gate *r'*_*t*_*,* Update Gate *z'*_*t*_*,* Candidate Activation *h*_*t*_*,* Hidden State *h'*_*t*_.

The forward and backwards hidden states at each time step, *h*_*t*_ and *h'*_*t,*_ respectively, are concatenated to create the Bi-GRU model’s final output. This output can then be processed further, such as passing it through a fully connected layer, to obtain the final prediction.

In summary, the Bi-GRU model incorporates two GRU layers: forward processing and backward processing of the input sequence. The equations provided an update on the hidden states of the GRU layers based on the input sequence and the model’s weights and biases. The forward and backward hidden states are combined to form the final output of the Bi-GRU model, enabling it to capture dependencies in both directions and make predictions based on the sequential data.

#### Bee colony optimization (BCO)

Several equations and algorithms are used in the Bee Colony Optimization algorithm to optimise the variables and configurations of a trained model. The fitness function, which assesses the quality of a solution based on the model’s performance on a specific task or dataset, is one of the critical equations used in BCO^42^. For example, the fitness function can be adjusted to the particular issue to maximise precision or minimise inaccuracy. The exploration phase of the BCO algorithm, in which bees execute local searches by perturbing the model’s parameters or hyperparameters, is another crucial algorithm. Mathematical operations such as addition, subtraction, multiplication, or mutation are frequently used to achieve this disturbance. Through a system comparable to a waggle dance, the bees exchange information about the quality of their solutions with one another. This information exchange makes the successful exploration and exploitation of various locations in the solution space possible. The best solutions are chosen during the exploitation phase based on their fitness values, and these solutions are used as the foundation for creating new solutions throughout the following iterations. Based on the problem domain and optimisation objectives, the particular equations and techniques employed in BCO can be tailored, resulting in practical parameter tuning and enhanced model performance.

1. *Initialisation* Assume that *N* is the population size and each bee represents a potential model solution with a set of hyperparameters or parameter configurations.
2. *Fitness Function* A solution *S'*_*s*_ quality is assessed by the fitness function, denoted by the symbol f_S._
3. *Local Search (Exploration Phase)* Local search involves perturbing the model’s parameters or hyperparameters. Assume *P* is a perturbation function and *S* is the solution.
4. $S{\prime} = S + P$, where *P* is a random value within a given range, is the parameter perturbation.
5. $S{\prime} = S * P$, where *P* is a random number within a given range, is the hyperparameter perturbation.

Information Sharing: Bees talk to one another by exchanging details on the calibre of their solutions. *S*_*i*_ and *f*_*Si*_ should stand in for the solution and its fitness value for the *i*_*th*_ bee.

They are choosing the Best Solutions and Generating New Solutions (Exploitation Phase). During exploitation, the best solutions are chosen, and fresh solutions are created. In Roulette Wheel Selection, a solution’s *f*_*Si*_ fitness value concerning the fitness sum as a whole determines the likelihood of selecting it. Using crossover operators, new solutions are created by mixing the parameters of previously chosen solutions. Random changes are made to the parameters of chosen solutions to explore new areas of the solution space.

### Training

Bidirectional Long Short-Term Memory (Bi-LSTM) and Bidirectional Gated Recurrent Unit (Bi-GRU) models of recurrent neural networks (RNNs)^40,43^ were used in this study to classify protein sequences on datasets related to B-cells and SARS-CoV. The training procedure for each dataset was split into four steps, each integrating different techniques to improve the performance of the RNN models.

**Figure 1:**
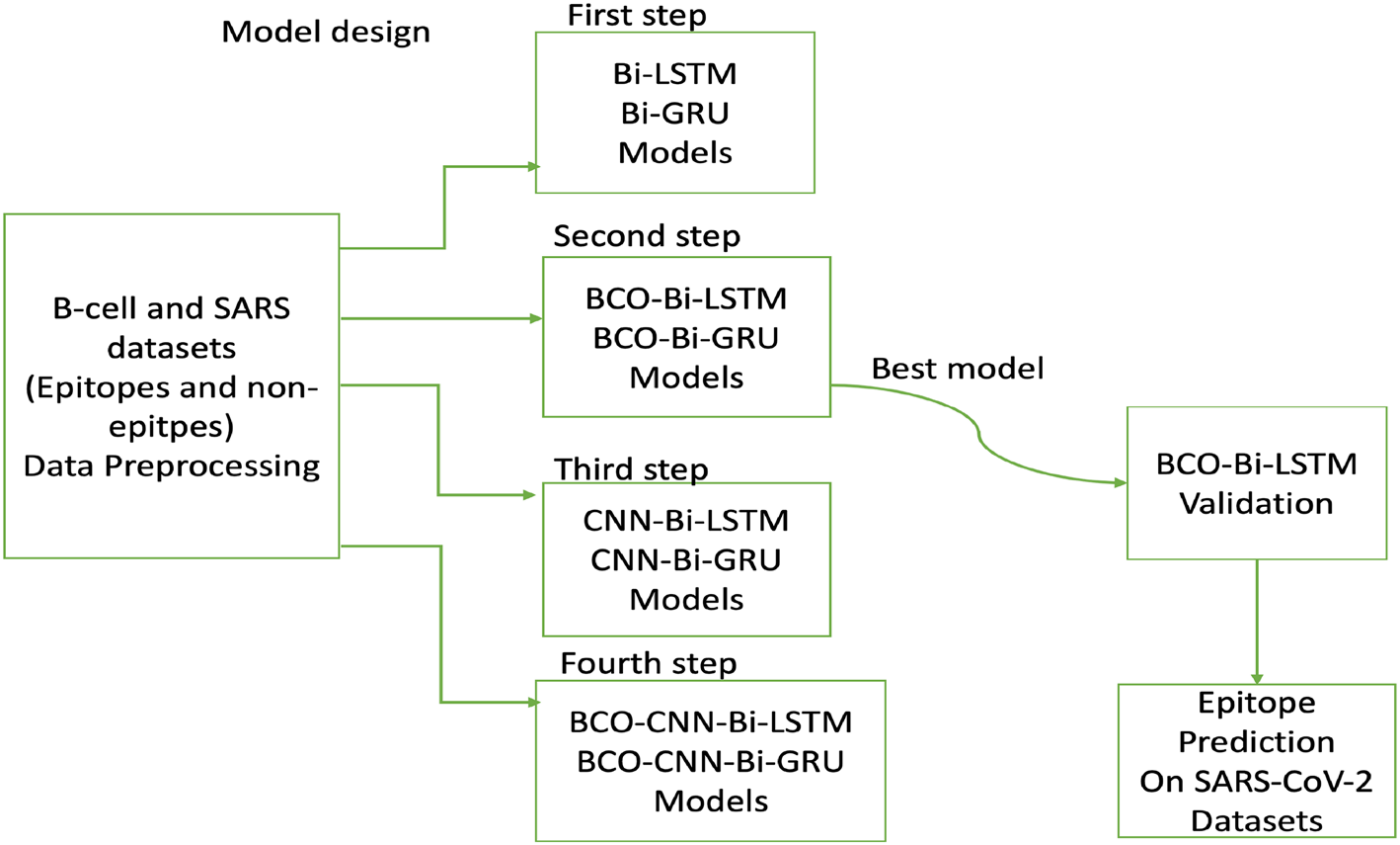
Proposed model designs. First, B-cell and SARS datasets were used to train the models in all four steps. Then, the best model was selected for the prediction of epitopes on the SARS-CoV-2 dataset.

It can be challenging to detect long-term dependencies because of the vanishing gradient problem, in which gradients get smaller and smaller throughout backpropagation. The Bi-LSTM and Bi-GRU models were individually trained in the first step. These models’ internal memory cells enable them to detect long-range relationships in sequential data. In the second stage, a hybrid approach combining Bi-LSTM and Bi-GRU with a method called Bee Colony Optimization (BCO) was used. BCO is an optimisation algorithm inspired by the behaviours of bees, as stated. It helps to fine-tune the parameters of the RNN models, improving their performance. Each standalone RNN model was optimised using BCO and trained separately (see Fig. 1).

The third step involved the addition of a Convolutional Neural Network (CNN)^44^ together with Bi-LSTM and Bi-GRU. Deep, feed-forward neural networks called CNNs can learn hierarchical spatial representations without the aid of manually created feature sets. Convolutional layers were used to extract pertinent local attributes from the input data using CNNs applied to each Bi-LSTM and Bi-GRU model. In the last phase, Bi-LSTM and Bi-GRU connected with BCO-CNN, previously paired with CNN (BCO-CNN). This strategy aimed to improve each of the models’ performance further by combining the benefits of BCO and CNN approaches.

After the input layer, an embedding layer was used to handle the input features effectively. The parameters of the embedding layer are carefully chosen to minimise training error, and it maps the input features to a higher-dimensional space. Several strategies were used during the training to enhance the model’s generalizability and avoid overfitting. The vanishing gradient problem was addressed by adding batch normalisation layers before the CNN layer, which speeds up training. Early stopping with a patience of 3 was used to end training when the accuracy or loss stopped increasing, and a dropout layer was used to prevent overfitting.

**Figure 2:**
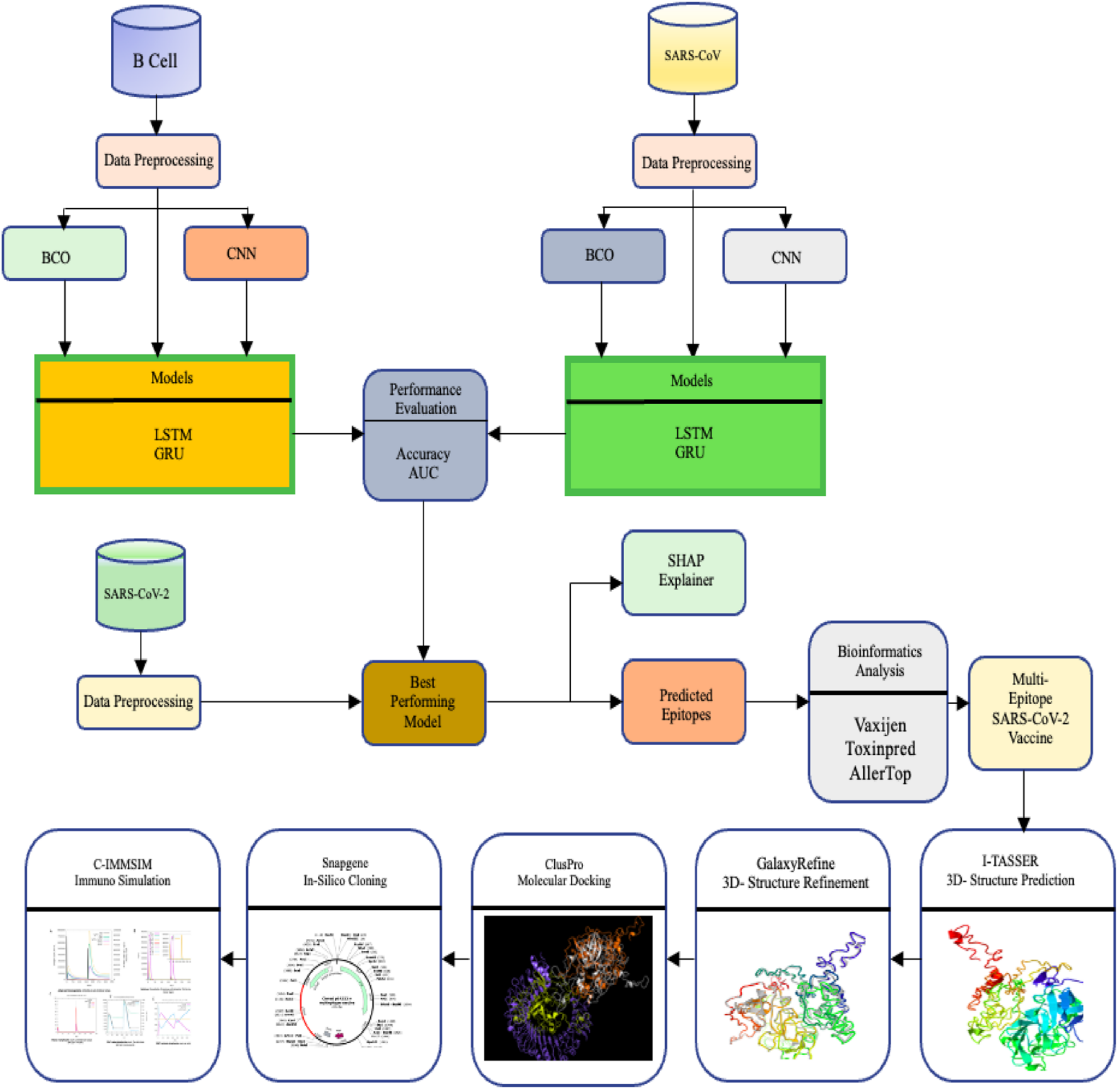
Process flow of the study.

The study aimed at epitopes that might be used in vaccine development using the SARS-CoV-2 datasets. The necessary bioinformatics tools were used to analyse the identified epitopes further. ToxinPred^45^ for toxicity verification, AllerTOP2.0^46^ for potential allergens in the predicted epitopes and VaxiJen^47^ for recognising antigens from the predicted epitopes. The proposed method and the overall workflow of the study can be visualised in Fig. 2,

#### SHAP approach

The deep black box model’s predictions are explained using the SHAP approach, also known as Shapely Additive Explanations. The average percentage of each feature’s contribution to the prediction value is mathematically known as the shapely value for that feature. This may be calculated by comparing the prediction value with and without the feature in each scene where it might interact with other features, then taking the average of those contributions as the SHAP value for that feature. The process is repeated for all other features to find their respective contributions using the Shapley kernel^48,49^. The analysis made use of the SARS-CoV-2 dataset. The contribution of every feature in the peptide sequences identified as epitopes or not was examined.

With this approach, the model must be trained on every feature subset ${\text{S}} \subseteq {\text{F}}$, where F is the collection of complete features. Every feature is given a significance value that reflects the impact of incorporating that component on the model’s prediction. Two models, f_S∪{i}_ and f_S_ are trained with the feature present and hidden, respectively, and are used to calculate the effect. Then, based on the current input ${\text{f}}_{{{\text{S}} \cup \left\{ {\text{i}} \right\}}} ({\text{x}}_{{{\text{S}} \cup \left\{ {\text{i}} \right\}}} ) - {\text{fS}}\left( {{\text{xS}}} \right)$, the projected outcomes from the two models are compared, in which ${x}_{S}$ represent the values of the input features in the set S. The differences mentioned above are calculated for all potential subsets S ⊆  F\{i} since the effect of omitting a feature relies on other characteristics in the model. Once calculated, the Shapley values are applied as feature contributions. The weighted average of all potential differences makes up these outcomes^48^.

$$\Phi {\text{i}} = \mathop \sum \limits_{{{\text{S}} \subseteq {\text{F}}\left\{ {\text{i}} \right\} }} \frac{{\left| S \right|!\left( {\left| F \right| - \left| S \right| - 1} \right)! }}{\left| F \right|}\left[ {f_{{S \cup \left\{ i \right\}}} \left( {x_{{S \cup \left\{ i \right\}}} } \right) - f_{S} \left( {x_{S} } \right)} \right]$$

### Investigation of allergenicity and antigenicity

Using the VaxiJen 2.0 server, the antigenicity of the finished vaccine will be evaluated (http://www.ddg-pharmfac.net/vaxijen/VaxiJen/VaxiJen.html)^47^. VaxiJen is the first service to forecast tumour, viral, and bacterial protective antigens without considering alignment. The models on the server were produced by employing auto-covariance (ACC) to pre-process amino acid properties. To evaluate allergens from the anticipated epitopes, the AllerTOP2.0 server will be used^46^. The server was created to provide alignment-independent models for allergen identification based on the fundamental chemical characteristics of the sequences of amino acids (https://www.ddg-pharmfac.net/AllerTOP/).

### Evaluation of toxicity and biochemical characteristics

Support vector machine (SVM) technology was used to construct the ToxinPred^45^ tool, which was used for the evaluation, and a score of 0.0 is considered non-toxic. To identify epitopes as poisonous or not, the ToxinPred score considers physiochemical characteristics of the epitope, such as molecular weight, hydrophilic nature, and potential mutations (https://webs.iiitd.edu.in/raghava/toxinpred/). The physicochemical properties of the final vaccination will be predicted using the ExPASy ProtParam server^50^. The physicochemical aspects include the half-life and instability index (https://web.expasy.org/protparam/).

### Multi-epitope BLAST screening

A BLAST was used to assess protein similarity to human proteins and lower the likelihood of autoimmunity. The UniProtKB Human database received the vaccine sequence for the blast investigation (https://www.ebi.ac.uk/Tools/sss/ncbiblast/). Moreover, with the use of the Pipeline Builder for Identification of Drug Targets (PBIT) (http://www.pbit.bicnirrh.res.in), we submitted the vaccine protein against the proteome of frequently occurring microbes of the gut considering the role the microorganisms play in safeguarding health. Proteins with an e-value threshold greater than 0.005 and less than 50% of a given sequence shared with the intestinal microbiome proteome were deemed non-homologous^51^. The final vaccine consists of a 50S ribosomal protein L2^52^ adjuvant for improving antigenicity (accession no. AXI95322.1) joined to the amino (N) terminus of the multi-subunit sequence by an EAAAK linker^53^. GPGPG linkers link ten B-cell epitope subunits together. A 6xHis tag is added at the C-terminal to facilitate protein purification and identification^54^.

### Prediction of secondary structure and solubility

The PSIPRED online programme (http://bioinf.cs.ucl.ac.uk/psipred/) was used to generate the secondary structures of the vaccine structure. It is an online server secondary structure generation tool that uses two feed-forward neural networks to predict protein structure. Furthermore, it effectively predicts transmembrane helix, fold, transmembrane topology, and domain recognition, among other things^55^. Additionally, the Protein–Sol server (https://protein-sol.manchester.ac.uk) employed a population average (PopAvrSol) of 0.45 to assess the solubility of a multi-epitope vaccine, with values greater than 0.45, suggesting improved solubility. The predicted scaled solubility value (QuerySol) of the protein will determine how soluble it is^56^.

### Predicting tertiary structures

The vaccine’s tertiary or three-dimensional (3D) model was made using the homology modelling program I-TASSER (Iterative Threading Assembly Refinement) platform (https://seq2fun.dcmb.med.umich.edu//I-TASSER/). It is an integrated platform for computational protein structure and function prediction based on the sequence, structure, and function approach. It leverages the Protein Data Bank (PDB) to find similar structural patterns^57^.

### Tertiary structure refinement

Using the GalaxyRefine web server (http://galaxy.seoklab.org/cgi-bin/submit.cgi?type=REFINE), the vaccine peptide’s 3D model will be enhanced. Based on refining techniques that were successfully tested in CASP10-based refinement studies, the GalaxyRefine server was created, and the structure’s relaxation was accomplished by repacking and molecular dynamics modelling. When applied to modern protein structure prediction models, this method can improve the overall standard of local and global structures^58^. The Molprobity score, GDT-HA score, RMSD score, and Clash score are used to assess the quality of the revised model.

### Immune receptors and the vaccine’s docking

A widespread tool for protein–protein docking, ClusPro (https://cluspro.bu.edu) was utilised for molecular docking. After docking with each combination of energy parameters, ten models are generated and clustered around populations of low-energy docked structures^59^. TLR4 with PDB ID: 2Z63 is the immunological receptor of choice. The docked unit with the lowest energy is picked among the ten ratings. PyMOL was used to visualise the 3D structure of the key interacting residues.

### Evaluation of codon adaptation and cloning

JCat (http://www.jcat.de) is an innovative approach for increasing protein output by identifying and optimising the codons of the target gene to adapt to different sequenced prokaryotes and particular hosts for eukaryotic gene expression. The process of optimising a particular sequence is split into two steps by JCat. First, the sequence is checked to determine if it matches a recognised amino acid or gene sequence. A series of amino acids is then translated from it. The codons with the highest relative adaptiveness for the pertinent amino acid for expression in the host are used to turn the amino acid sequence into a gene sequence in a subsequent step^60^. Next, the vaccine’s codons will be cloned into the pRSFDuet-1 vector using the Snapgene application to achieve the most significant possible expression within *E. coli* (available at (https://www.snapgene.com/free-trial/ ).

### Polymerase chain reaction with agarose gel electrophoresis simulation

Using SnapGene, the primers were designed according to the Tm value and the length. The typical primer length is 20–23 bp, the Tm value is selected at 55–65 °C, the annealing temperatures are 1 °C, the GC contents are around 50–65%, and a protective nucleobase is inserted at the 5′ end. Lastly, using SnapGene, the recombinant plasmid was used to simulate agarose gel electrophoresis (https://www.snapgene.com/free-trial/).

### Immune response to vaccine simulation

The vaccine immune response profile will be made available through the C-ImmSim internet simulation service. The vaccination triggers a comparable immune reaction when it enters the body as an antigen. C-ImmSim was utilised to replicate the magnitude and nature of immunological reactions triggered by the MEV in people. C-ImmSim will analyse a mammalian immune system’s humoral and cellular reaction after the vaccine’s initial booster dose. (https://kraken.iac.rm.cnr.it/C-IMMSIM/index.php?page=1)^61^.

## Results and discussion

The performance of several models on the SARS dataset is presented in Table 1 and is assessed in terms of accuracy and AUC (Area Under the ROC Curve). The Bi-LSTM model’s accuracy and AUC were 0.8462 and 0.8545, respectively. With the SARS dataset, the Bi-LSTM does an excellent job of accurately classifying the epitopes. The long-term dependencies in sequential data are well-represented by the LSTM architecture. The Bi-GRU model achieved an accuracy of 0.77 and an AUC of 0.894, even though it is noticeably less accurate than the Bi-LSTM model.

Applying Bee Colony Optimization improves the BCO-Bi-LSTM model’s performance with an AUC of 0.944 and an accuracy of 0.92. The BCO-Bi-LSTM model shows improved performance. It implies that the optimisation process helps in identifying optimal model configurations. The BCO-Bi-GRU model has an accuracy and an AUC of 0.8846. It works much less than BCO-Bi-LSTM, although it produces better results than the basic Bi-GRU model. It demonstrates that BCO optimisation can be favourable for GRU and LSTM architectures.

The BCO-CNN-Bi-LSTM and BCO-CNN-BI-GRU models had accuracy scores of 0.8 and 0.826, respectively, and AUC scores of 0.839 and 0.8864, respectively. See Table 2. Combining BCO and Convolutional Neural Networks (CNN) with either Bi-LSTM or Bi-GRU, these models provide competitive performance. With CNN layers added, the models can identify regional patterns and extract meaningful information from the input data.

**Table 2:** SARS dataset.

Models	Accuracy	AUC
Bi-LSTM	0.8462	0.8545
Bi-GRU	0.77	0.894
BCO-Bi-LSTM	0.92	0.944
BCO-Bi-GRU	0.8846	0.879
BCO-CNN-Bi-LSTM	0.8	0.839
BCO-CNN-BI-GRU	0.826	0.8864
CNN- Bi-GRU	0.817	0.6009
CNN- Bi-LSTM	0.8654	0.8854

The CNN-Bi-GRU model has an accuracy and an AUC of 0.817. Its performance is moderate when compared to the other models in the table. This implies that the combination of CNN and GRU may not be as effective for this dataset. Further research might be needed to improve performance, or the model might need to be modified. The accuracy and AUC of the CNN- Bi-LSTM model are 0.8654 and 0.8854, respectively. It performs admirably, much like the Bi-LSTM model. Accurate predictions are made due to the effective capture of spatial and temporal dependencies in the data by the CNN and LSTM combo.

The results show how effectively LSTM-based models, specifically Bi-LSTM and BCO-Bi-LSTM, classified the SARS dataset. CNN layers and BCO tuning improve performance even more. The performance of Bi-LSTM-based models is slightly superior to that of Bi-GRU models. These findings provide helpful direction for choosing the most appropriate models for SARS epitope prediction-related tasks. The outcomes from both tables show how well LSTM-based models, particularly LSTM, capture sequential patterns in the Bcell and SARS datasets. Additional layers like CNN and optimisation methods like Bee Colony Optimization can be added to increase performance even more. However, the lower accuracy and AUC scores suggest that the Bcell dataset is more challenging to capture. This implies that in order to get better results on the Bcell dataset, more investigation and model tuning could be necessary, as the results show in Table 3.

**Table 3:** BCell dataset.

Models	Accuracy	AUC
Bi-LSTM	0.744	0.709
Bi-GRU	0.741	0.7
BCO-Bi-LSTM	0.817	0.83
BCO-Bi-GRU	0.81	0.79
BCO-CNN-Bi-LSTM	0.79	0.664
BCO-CNN-Bi-GRU	0.787	0.6403
CNN-Bi-GRU	0.781	0.775
CNN- Bi-LSTM	0.79	0.8

### An explainable deep model with SHAP

**Figure 3:**
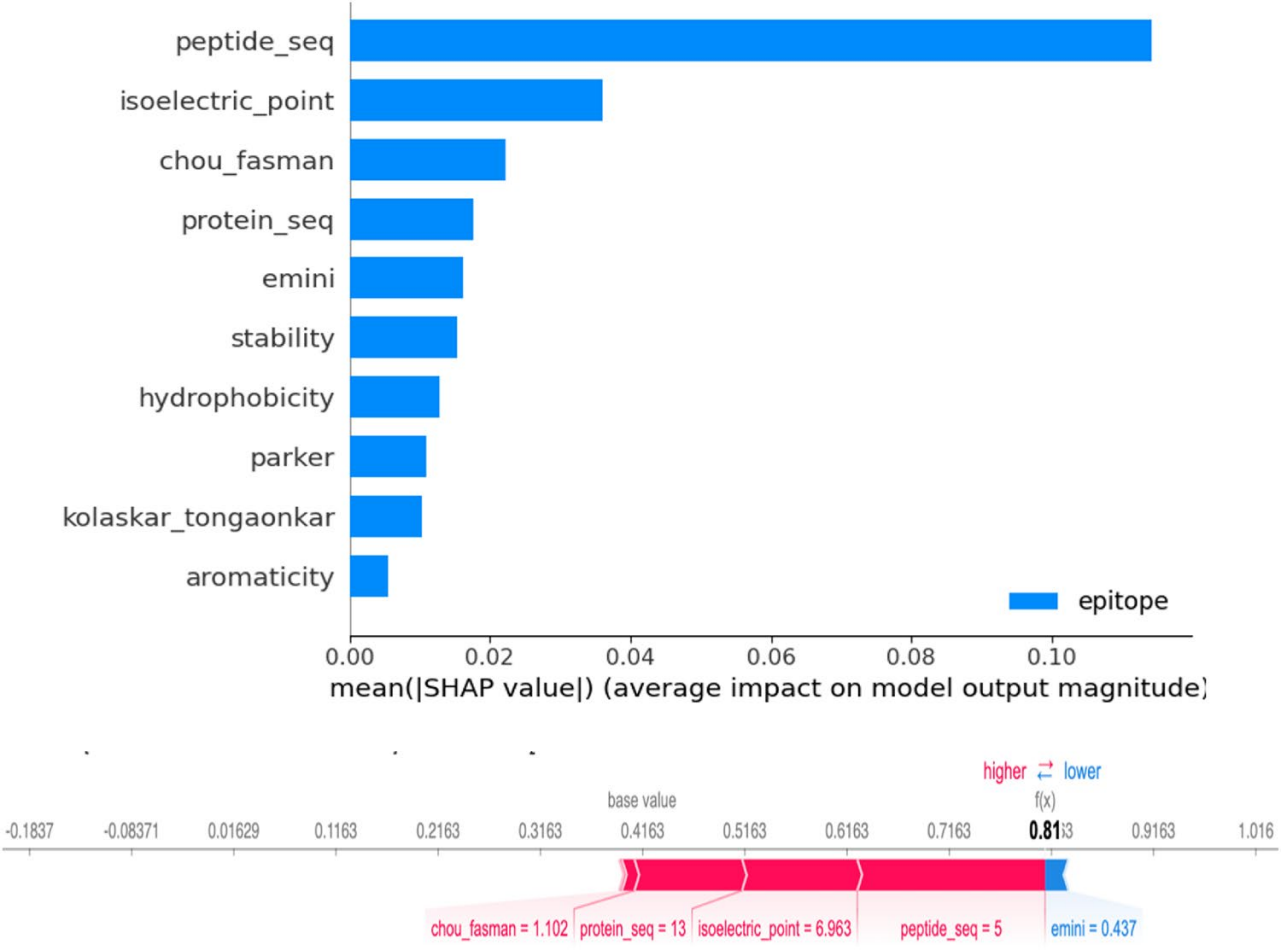
SHAP values plot for feature importance. The SHAP value’s magnitude reveals how significant a feature is to the model’s prediction. A more significant absolute SHAP value indicates a higher relevance. A positive SHAP value and vice versa show the contribution of a feature to a more excellent prediction value.

The deep models here may be thought of as “black boxes” because peptides are fed into the models, and predictions about whether or not they are epitopes are retrieved from the last layer without any explanation of the decision-making process. Shapley values were computed for each feature to understand each feature’s role in forecasting. Determining the average value contributed by every feature will assist in explaining why the black box model produced such forecasts. Lower magnitude numbers or negative values indicate less relevance in the forecast, and the more positive the SHAP value, the more significant it is in making the prediction^48^. Therefore, we learned that peptide sequence is crucial in the prediction task. This is very important since each peptide sequence has its component amino acids. Next is the isoelectric point, chou_fasman (beta turn), protein sequence, and relative surface accessibility or emini see Fig. 3.

The prediction task’s most crucial element was determined to be the peptide sequence. This suggests that whether a peptide is categorised as an epitope or not is strongly influenced by the exact arrangement of amino acids inside it. The model probably picked up peptide sequence motifs or patterns pointing to epitopes. As a result, different amino acid combinations or variants play a substantial role in the model’s choice-making process. Identifying epitopes also requires consideration of the isoelectric point. The pH level where the peptides’ net charges are zero is known as the isoelectric point. According to the feature’s positive SHAP value, epitope prediction may be positively influenced by particular isoelectric point ranges or values. It suggests that the peptide’s charge distribution affects the strength of its ability to impact an epitope. Next, an important characteristic is the Chou_Fasman score, notably the Beta Turn conformation. The fact that this feature has a positive SHAP value shows that specific motifs or traits connected to beta turn conformations are predictive of epitopes. The model probably discovered that secondary structural patterns or particular combinations of amino acids are connected to beta turns and help predict epitopes. In addition to the peptide, the protein sequence also plays a role in the prediction process. It captures the larger context of the protein the peptide belongs to, even if it can contain information that matches the peptide sequence. The model could have picked up on certain features or patterns in the protein sequence that help predict epitopes. Another essential element that Emini refers to is thought to be relative surface accessibility. The information this characteristic provides describes the amino acids exposed to or readily available on the protein’s surface. The fact that this characteristic has a positive SHAP value shows that the prediction of epitopes may be affected by specific accessibility rates or combinations of exposed amino acids. The model probably discovered that, in contrast to non-epitopes, epitopes frequently have distinctive surface properties.

In general, the interpretation of the SHAP data emphasises the crucial elements that help the model forecast epitopes. Lower magnitude or negative SHAP values imply less relevance, whereas positive SHAP values highlight the significance of particular feature values or patterns in the forecast. By providing insights into the underlying mechanisms influencing the predictions, this knowledge aids in understanding how and why the black box model generated its predictions.

### Allergenicity and antigenicity assessment

The Vaxijen 2.0 internet server grades the resulting multi-epitope vaccine sequence’s antigenicity. We assess each vaccine component, including the adjuvant, for its antigenicity (see Table 4). With a threshold of 0.4, the peptide is said to be an antigen, according to the Vaxijen tool. The Vaxijen score for the entire final vaccine is 0.8772, indicating that our final vaccine has a high level of antigenicity. ANTIGENpro was further utilised to verify the predictions made by the Vaxijen tool (see Table 4), and it confirmed the vaccine’s antigenicity with a 0.8959 prediction score. AllerTOP 2.0 server forecast that the finished vaccine and each of its parts and adjuvant will be allergy-free (see Table 5).

**Table 4:** Predictions of Vaxijen and ANTIGENpro tools for antigenicity.

Peptide	Vaxijen Prediction	Vaxijen Score	ANTIGENpro	ANTIGENpro score
SYQTQTNSPSGAGSVASQ	Antigen	1.4888	Antigen	0.831015
VYDPLQPELDSFKEELDK	Antigen	0.4309	Antigen	0.041408
GKYEQYIKGSGRENLYFQ	Antigen	0.5567	Antigen	0.258093
GYIPEAPRDGQAYVRKDGE	Antigen	0.5032	Antigen	0.270398
EYVSQPFLMDLEGKQGN	Antigen	1.2111	Antigen	0.102666
EKGIYQTSNFRVQPTES	Antigen	0.7705	Antigen	0.834126
TSNFRVQPTESIVRFPN	Antigen	0.5719	Antigen	0.421434
IAPGQTGKIADYNYKLP	Antigen	0.8528	Antigen	0.328423
DSKVGGNYNYLYRLFRK	Antigen	0.7783	Antigen	0.067362
DQLTPTWRVYSTGSNVF	Antigen	0.7793	Antigen	0.648966
Multi-epitope vaccine	Antigen	0.8772	Antigen	0.895861
Adjuvant	Antigen	0.7653	Antigen	0.820472

**Table 5:** Allertop tool results for allergenicity screening.

Peptide	Prediction	The nearest protein
SYQTQTNSPSGAGSVASQ	Probable non-allergen	UniProtKB accession number Q15517
VYDPLQPELDSFKEELDK	Probable non-allergen	UniProtKB accession number P02647
GKYEQYIKGSGRENLYFQ	Probable non-allergen	UniProtKB accession number Q8TAP6
GYIPEAPRDGQAYVRKDGE	Probable non-allergen	UniProtKB accession number Q96LW7
EYVSQPFLMDLEGKQGN	Probable non-allergen	UniProtKB accession number A6MZC4
EKGIYQTSNFRVQPTES	Probable non-allergen	UniProtKB accession number O04437
TSNFRVQPTESIVRFPN	Probable non-allergen	UniProtKB accession number A8QPS0
IAPGQTGKIADYNYKLP	Probable non-allergen	UniProtKB accession number Q6RJU6
DSKVGGNYNYLYRLFRK	Probable non-allergen	UniProtKB accession number Q2XPP4
DQLTPTWRVYSTGSNVF	Probable non-allergen	UniProtKB accession number Q40161
Multi-epitope vaccine	Probable non-allergen	UniProtKB accession number P46379
Adjuvant	Probable non-allergen	UniProtKB accession number Q8TEP8

### Toxicity and biochemical characteristics

Vaccine safety is a priority. Therefore, it must not have a high potential for toxicity, and its physicochemical characteristics must also be considered when assessing its interactions with biological systems^62^. The toxinPred server is used to forecast toxicity. The findings of our examinations of each subunit’s physicochemical and toxicological characteristics are displayed in Table 6. Each subunit was found to be safe. Therefore, there are no hazardous component peptides in either vaccine.

**Table 6:** ToxinPred results for toxicity analysis.

Peptide sequence	SVM score	Toxicity	Hydrophobicity	Hydropathicity	Hydrophilicity	Molecular weight (Da)
SYQTQTNSPSGAGSVASQ	− 0.97	Non-Toxin	− 0.17	− 0.85	− 0.18	1770.05
VYDPLQPELDSFKEELDK	− 1.3	Non-Toxin	− 0.24	− 1.08	0.55	2328.83
GKYEQYIKGSGRENLYFQ	− 0.6	Non-Toxin	− 0.28	− 1.37	0.16	2180.68
GYIPEAPRDGQAYVRKDGE	− 1.1	Non-Toxin	− 0.31	− 1.32	0.65	2121.56
EYVSQPFLMDLEGKQGN	− 1.44	Non-Toxin	− 0.15	− 0.76	0.1	1955.43
EKGIYQTSNFRVQPTES	− 2.11	Non-Toxin	− 0.29	− 1.22	0.25	1984
TSNFRVQPTESIVRFPN	− 1.72	Non-Toxin	− 0.24	− 0.63	− 0.02	1992.46
IAPGQTGKIADYNYKLP	− 0.67	Non-Toxin	− 0.1	− 0.54	− 0.12	1849.37
DSKVGGNYNYLYRLFRK	− 1.35	Non-Toxin	− 0.32	− 1.07	0.07	2093.62
DQLTPTWRVYSTGSNVF	− 1.44	Non-Toxin	− 0.13	− 0.46	− 0.42	1971.4

ExPASy ProtParam Tool also predicts molecular weight, hydropathicity, charge, half-life, instability index, and pI (theoretical isoelectric point value). The completed vaccine’s estimated hydropathicity value is − 0.812. This low number suggests that our final vaccine will be hydrophilic and efficiently bind to water molecules^17^. The finalised vaccine’s half-life is anticipated at 30 in vitro hours, while for in vivo, it is more than 20 h. Our final vaccine is stable since the expected Instability Index is 29.69, which is below the cut-off of 40. With a calculated pI of 9.80, the finished vaccine is highly basic and alkaline. The molecular mass of the finished vaccine is found to be 54880.72 Da.

### Homology analysis and the assembling of a finalised multi-epitope vaccine candidate

**Figure 4:**
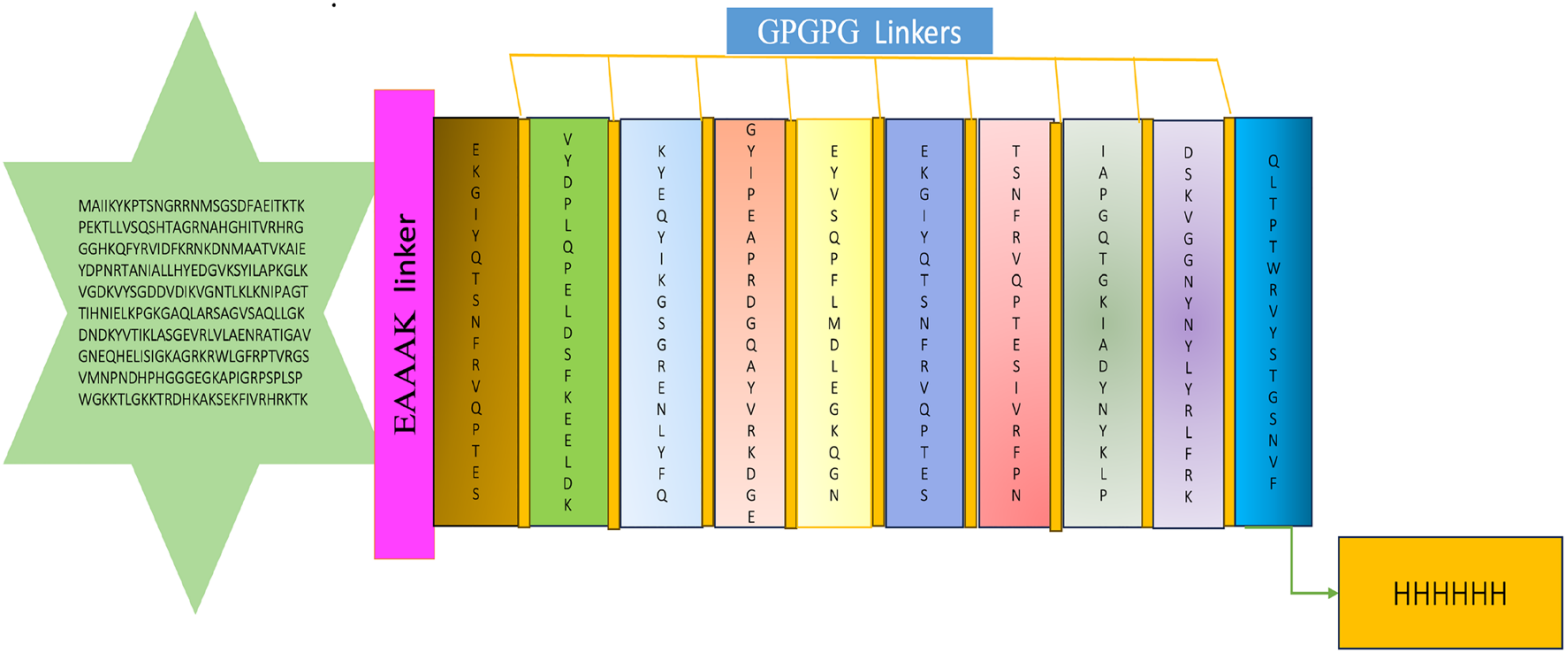
Multi-epitope vaccine schematic diagram. The multi-epitope vaccine sequence, which is 509 amino acids long, links an adjuvant (green) at the N-terminal end with the multi-epitope sequence using an EAAAK linker (purple). GPGPG linkers (yellow) were used to link ten epitopes at the tail, a poly His tag was lastly inserted.

We conduct a BLAST search on all 11 vaccine subunits utilising the Uniprot database to rule out probable autoimmunity. A subunit with more than 35% identity with the human proteome will be regarded as a homologous protein. None of the 11 vaccine components we ultimately decided to use in the vaccine production exhibits significant similarity with the human proteome. See Table 7 for results. The 50S ribosomal protein L7/L12 showed significant identity with the multi-epitope vaccine compared to the gut microbiota during the search for similarities, while other components did not show any homology. To improve the immune response, the finished vaccine contains an adjuvant, 50S ribosomal protein L2 (accession no. AXI95322.1), which is joined to the amino (N) terminum of the multi-subunit sequence utilising the EAAAK linker. There are 509 amino acid residues in the completed vaccine. 10 B-cell epitope subunits are fused via GPGPG linkers^53^ see Fig. 4. To aid in the purification and identification of the protein, eventually, the C-terminal is tagged with a 6xHis tag^54^.

**Table 7:** BLAST screening results against UniProtKB Human database.

	Protein	Organism name	Score (Bits)	Identities %	E-value
1	50S ribosomal protein L2	*Lacticaseibacillus paracasei*	550.1	96.4	0.0
2	50S ribosomal protein L2	*Lacticaseibacillus casei*	550.1	96.4	0.0
3	50S ribosomal protein L2	*Latilactobacillus sakei*	447.2	77.7	1.2E−155
4	50S ribosomal protein L2	*Lactiplantibacillus plantarum*	437.2	78.6	1.2E−151
5	50S ribosomal protein L2	*Lactobacillus helveticus*	436.8	77.5	1.6E−151
6	50S ribosomal protein L2	*Ligilactobacillus salivarius*	436.0	78.4	3.1E−151
7	50S ribosomal protein L2	*Lactobacillus johnsonii*	433.0	76.4	5.3E−150
8	50S ribosomal protein L2	*Lactobacillus acidophilus*	432.6	76.8	7.5E−150
9	50S ribosomal protein L2	*Leuconostoc citreum*	430.6	77.3	4.1E−149
10	50S ribosomal protein L2	*Lactobacillus gasseri*	430.6	76.1	4.3E−149
11	50S ribosomal protein L2	*Levilactobacillus brevis*	427.9	77.0	5.7E−148
12	50S ribosomal protein L2	*Lactobacillus delbrueckii* (strain ATCC BAA-365/Lb-18)	426.4	74.6	2.0E−147
13	50S ribosomal protein L2	*Lactobacillus delbrueckii* (strain ATCC 11842/DSM 20081/BCRC 10696/JCM 1002/NBRC 13953/NCIMB 11778/NCTC 12712/WDCM 00102/Lb 14)	426.4	74.6	2.0E−147
14	50S ribosomal protein L2	*Pediococcus pentosaceus*	422.2	76.1	1.1E−145
15	50S ribosomal protein L2	*Leuconostoc mesenteroides*	421.8	75.5	1.3E−145
16	50S ribosomal protein L2	*Limosilactobacillus reuteri*	419.9	76.1	8.3E−145
17	50S ribosomal protein L2	*Limosilactobacillus reuteri*	419.9	76.1	8.3E−145
18	50S ribosomal protein L2	*Limosilactobacillus fermentum*	414.5	75.0	1.1E−142
19	50S ribosomal protein L2	*Oenococcus oeni*	412.9	74.6	4.1E−142
20	50S ribosomal protein L2	*Enterococcus faecalis*	410.6	73.1	3.0E−141
21	50S ribosomal protein L2	*Macrococcus caseolyticus*	401.7	71.7	9.1E−138
22	50S ribosomal protein L2	*Streptococcus thermophilus* (strain ATCC BAA-491/LMD-9)	400.6	69.8	2.7E−137
23	50S ribosomal protein L2	*Streptococcus thermophilus* (strain ATCC BAA-250/LMG 18311)	400.6	69.8	2.7E−137
24	50S ribosomal protein L2	*Streptococcus thermophilus* (strain CNRZ 1066)	400.6	69.8	2.7E−137
25	50S ribosomal protein L2	*Streptococcus uberis*	400.2	68.7	3.8E−137
26	50S ribosomal protein L2	*Streptococcus pneumoniae* (strain Taiwan19F-14)	398.7	68.0	1.5E−136
27	50S ribosomal protein L2	*Streptococcus pneumoniae* (strain P1031)	398.7	68.0	1.5E−136
28	50S ribosomal protein L2	*Streptococcus pneumoniae* (strain JJA)	398.7	68.0	1.5E−136
29	50S ribosomal protein L2	*Streptococcus pneumoniae* (strain ATCC BAA-255/R6)	398.7	68.0	1.5E−136
30	50S ribosomal protein L2	*Streptococcus pyogenes* (strain NZ131)	398.7	69.1	1.5E−136
31	50S ribosomal protein L2	*Streptococcus pneumoniae* (strain CGSP14)	398.7	68.0	1.5E−136
32	50S ribosomal protein L2	*Streptococcus pyogenes* (strain SSI-1)	398.7	69.1	1.5E−136
33	50S ribosomal protein L2	*Streptococcus pneumoniae* (strain ATCC BAA-334/TIGR4)	398.7	68.0	1.5E−136
34	50S ribosomal protein L2	*Streptococcus pyogenes* (strain MGAS6180)	398.7	69.1	1.5E−136
35	50S ribosomal protein L2	*Streptococcus pneumoniae* (strain ATCC 700669/Spain 23F-1)	398.7	68.0	1.5E−136
36	50S ribosomal protein L2	*Streptococcus pneumoniae* (strain Hungary19A-6)	398.7	68.0	1.5E−136
37	50S ribosomal protein L2	*Streptococcus pyogenes* (strain Manfredo)	398.7	69.1	1.5E−136
38	50S ribosomal protein L2	*Streptococcus pyogenes* (strain MGAS10270)	398.7	69.1	1.5E−136
39	50S ribosomal protein L2	*Streptococcus pyogenes* (strain MGAS9429)	398.7	69.1	1.5E−136
40	50S ribosomal protein L2	*Streptococcus pyogenes* (strain MGAS2096)	398.7	69.1	1.5E−136
41	50S ribosomal protein L2	*Streptococcus pyogenes* (strain MGAS8232)	398.7	69.1	1.5E−136
42	50S ribosomal protein L2	*Streptococcus pneumoniae* (strain 70585)	398.7	68.0	1.5E−136
43	50S ribosomal protein L2	*Streptococcus pyogenes* (strain ATCC BAA-946/MGAS10394)	398.7	69.1	1.5E−136
44	50S ribosomal protein L2	*Streptococcus pneumoniae* (strain G54)	398.7	68.0	1.5E−136
45	50S ribosomal protein L2	*Streptococcus pyogenes* (strain ATCC BAA-595/MGAS315)	398.7	69.1	1.5E−136
46	50S ribosomal protein L2	*Streptococcus pneumoniae* (strain D39/NCTC 7466)	398.7	68.0	1.5E−136
47	50S ribosomal protein L2	*Streptococcus pyogenes* serotype M1	398.7	69.1	1.5E−136
48	50S ribosomal protein L2	*Streptococcus pyogenes* (strain MGAS10750)	398.7	69.1	2.2E−136
49	50S ribosomal protein L2	*Streptococcus zooepidemicus*	397.9	68.3	1E−136
50	50S ribosomal protein L2	*Streptococcus zooepidemicus* (strain MGCS10565)	397.9	68.3	1E−136

### Secondary structure and solubility prediction

**Figure 5:**
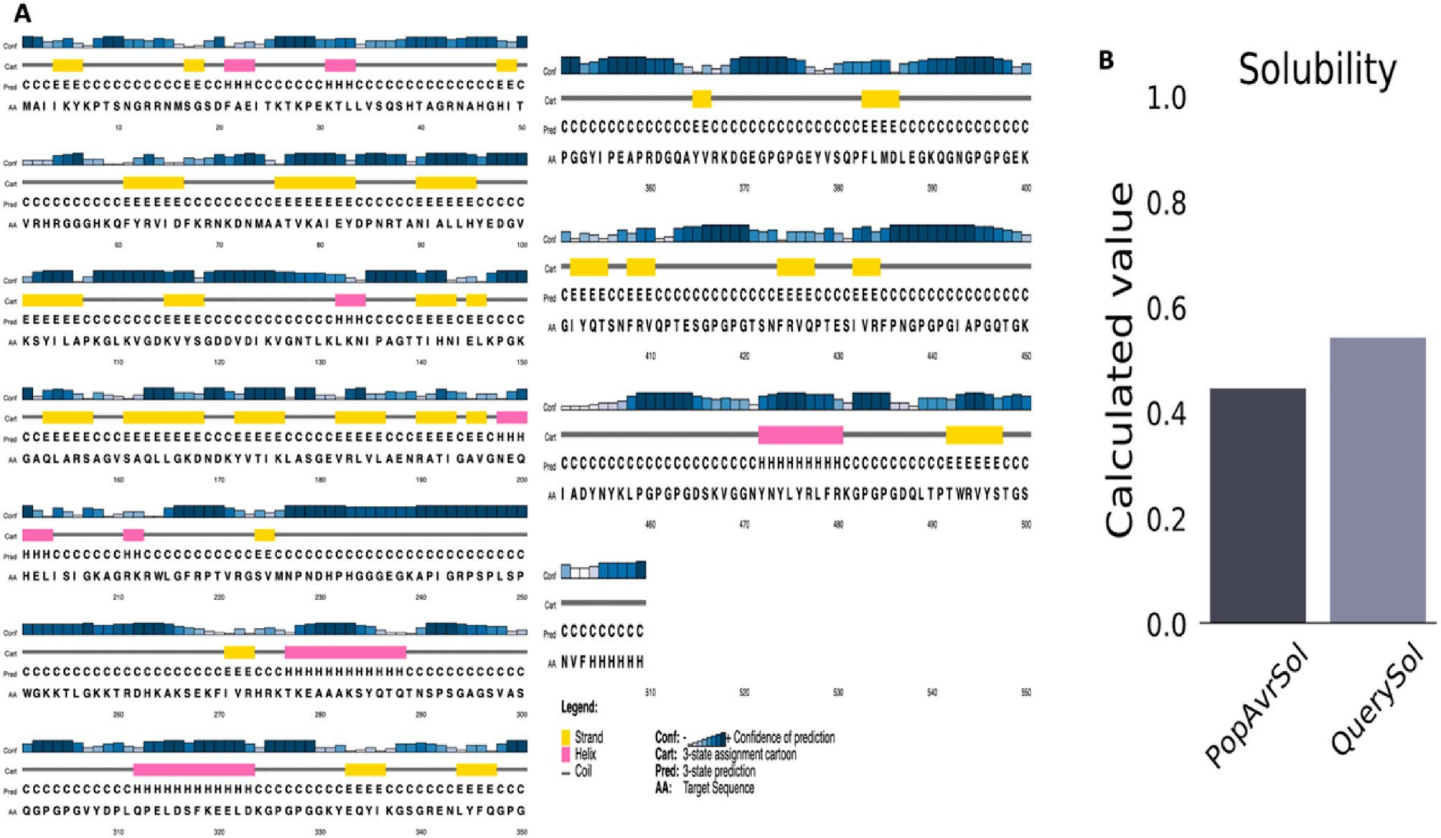
Predictions about the secondary structure of the vaccine construct and on solubility analysis. (**A**) The secondary structure prediction indicated that the likelihood of the protein forming antigenic epitopes is indicated by the high percentage of β strands 18% and random coil 73% seen in the MEV (**B**) ProtSol predicted the solubility value of 0.543.

The PSIPRED server was used to predict the secondary structure of the vaccine, which has 8.34% alpha helices, 18% beta strands, and 73.6% coils (see Fig. 5A). Finally, the vaccine was predicted to be soluble by the Sol-Pro tool. According to Fig. 5B, the estimated scale solubility value of 0.543 indicated good solubility because it was higher than the population average (PopAvrSol) of 0.45.

### A favoured vaccine’s multi-epitope 3D structure

**Figure 6:**
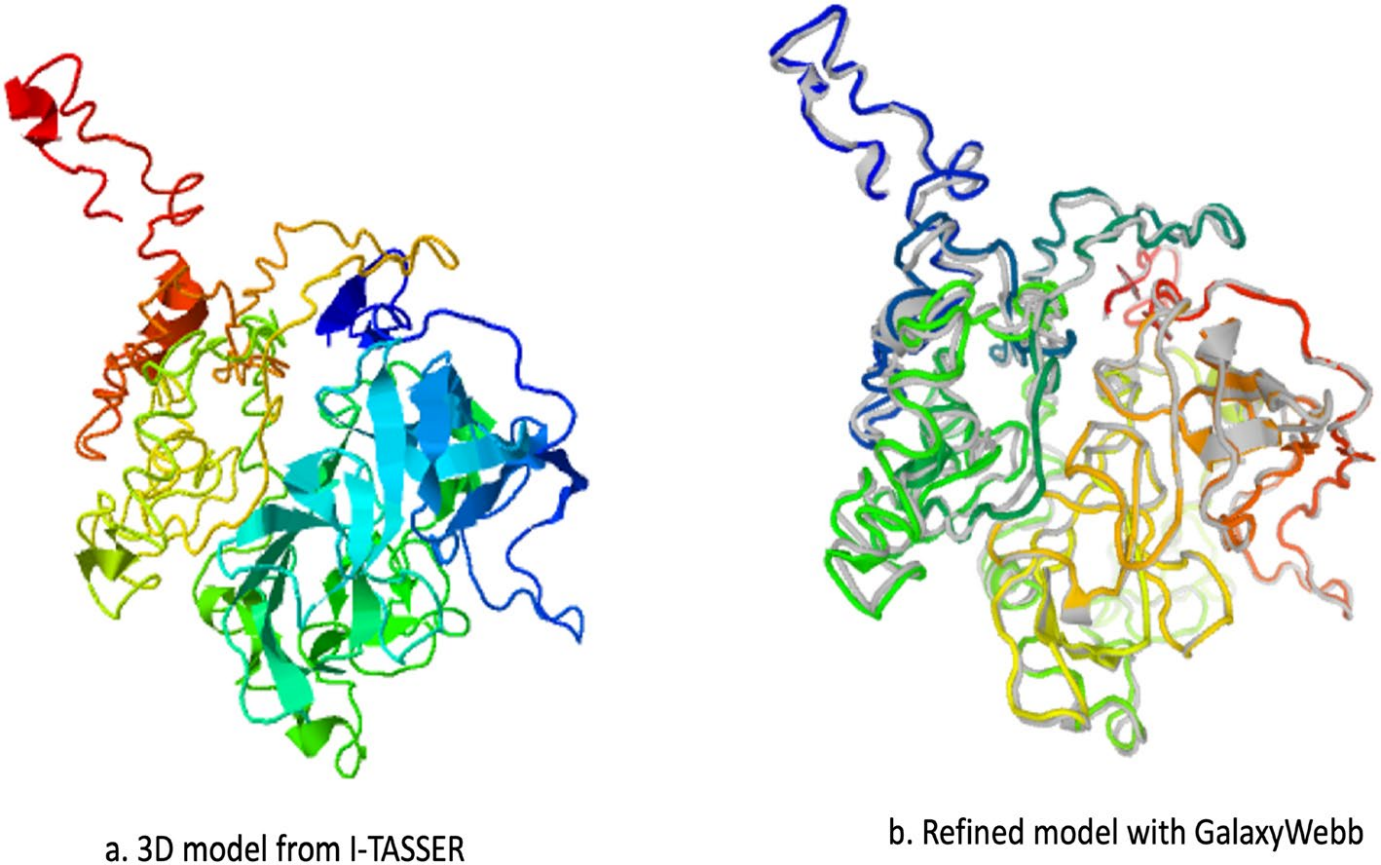
(**a**) A C-score of − 2.93 was assigned to I-TASSER’s 3D structural vaccine model. This number reveals that this 3D model is high quality (**b**) GalaxyWeb’s 3D model improvement.

The I-TASSER server begins modelling with structure templates located in the PDB database. Despite the server’s ability to generate thousands of template alignments, it only uses the best ones based on accuracy where Z-score > 1 = good alignment. The five likely tertiary structures associated with the multi-epitope vaccine were predicted using the top 10 templates, having Z-scores varying from 1.78 to 6.21. Each of these five models’ unique C-scores was − 2.93, − 3.79, − 2.58, − 3.84, and − 2.65. A proper global topology is indicated by a C-score value of > -1.5, which usually falls between − 5 and 2. We chose the multi-epitope tertiary structure with a C-score of − 2.93 because it is the first model since the first model usually has the best quality, as shown in Fig. 6a. Its RMSD is 14.6 ± 3.7 Å, and it has a TM-score of 0.38 ± 0.13. Figure 6b shows the refined structure. This shows that the tertiary structure model has good quality.

### Vaccine’s 3D structural refinement

The GalaxyWeb server then underwent a refining procedure to enhance the quality of the structure after selecting the best 3D model. The server then produced five improved models as a result of that. Model 3 was chosen based on the parameters. The GDT-HA of model 3 was 0.8811, close to the initial 3D model. RMSD for atomic distance score of 0.644 was the lowest, indicating that the model is the most stable. The MolProbity 2.636 is lower than the original, indicating a decrease in crucial errors. Clash score was 28.2, poor rotamers 2.0, and Rama’s score was 80.7. The refined structure details can be seen in Table 8.

**Table 8:** Models following improvement with the GalaxyWeb server.

Model	GDT-HA	RMSD	MolProbity	Clash score	Poor rotamers	Rama favoured
Original	1.0000	0.000	3.625	20.0	16.1	60.4
^ 1^Model	0.8698	0.659	2.634	26.4	0.7	80.9
^2^Model	0.8713	0.644	2.636	26.9	1.0	81.3
^3^Model	0.8811	0.647	2.889	28.2	2.0	80.7
^4^Model	0.8743	0.646	2.637	25.9	0.5	80.1
^5^Model	0.8708	0.674	2.611	24.5	1.0	80.3

**Figure 7:**
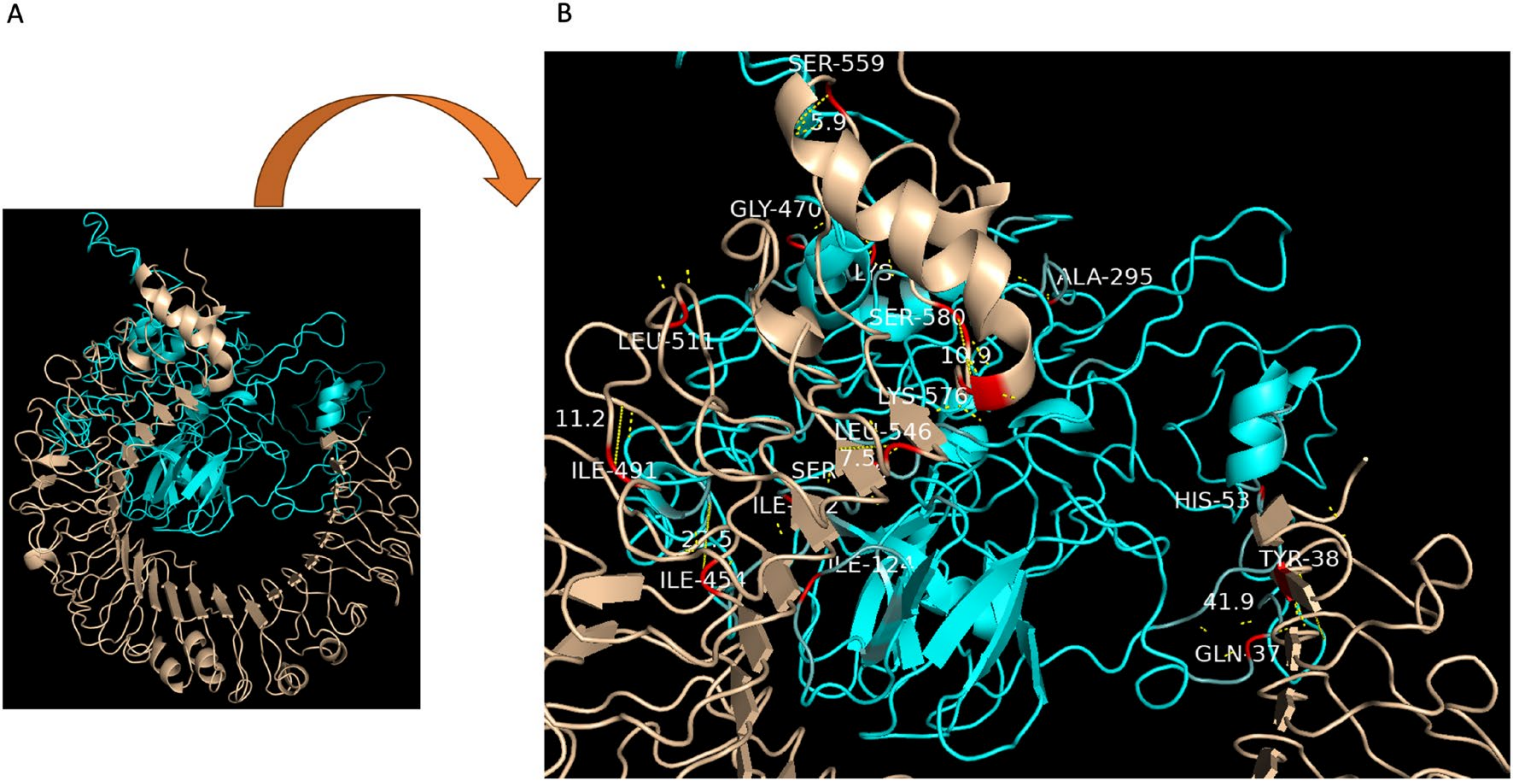
(**A**) Molecular docking depicts a ligand-receptor complex with the TLR4 (PDB ID: 2Z63) receptor and the multi-epitope vaccine as the ligand. The cyan colour represents the vaccine, and the brown represents the receptor. (**B**) Using the visualising programme PyMol, the interactions between residues in the MEV and TLR4 complex were examined, and their 3D image was captured.

The molecular docking technique can investigate the strength and binding capability of a docked complex among a ligand and receptor molecule. We choose to carry out the molecular docking on TLR4 as the immunological receptor since it is a crucial human protein enabling pathogen detection and immune response. The updated 3D model of our final vaccine and the immunological receptor TLR4 (PDB ID: 2Z63) are molecularly docked using the ClusPro 2.0, as shown in Fig. 7A. Of the various outcomes that ClusPro docking produces, the top 10 outcomes were chosen for examination. The best model docked was output number 6, demonstrating the best interactions between the receptor and ligand. Among all the docked models created, the one with the lowest energy score, 919.5, was selected, indicating that the vaccine has a strong affinity for the model and can successfully fill the receptor. PyMOL was used to visualise the three-dimensional structure interactions after choosing the optimal docking structure. The findings revealed the presence of numerous polar interactions between the vaccine and TLR4. The interacting residues can be seen in red, and their distance measured in Å was displayed as yellow dash lines in Fig. 7B.

### Adaptation of codons and cloning

**Figure 8:**
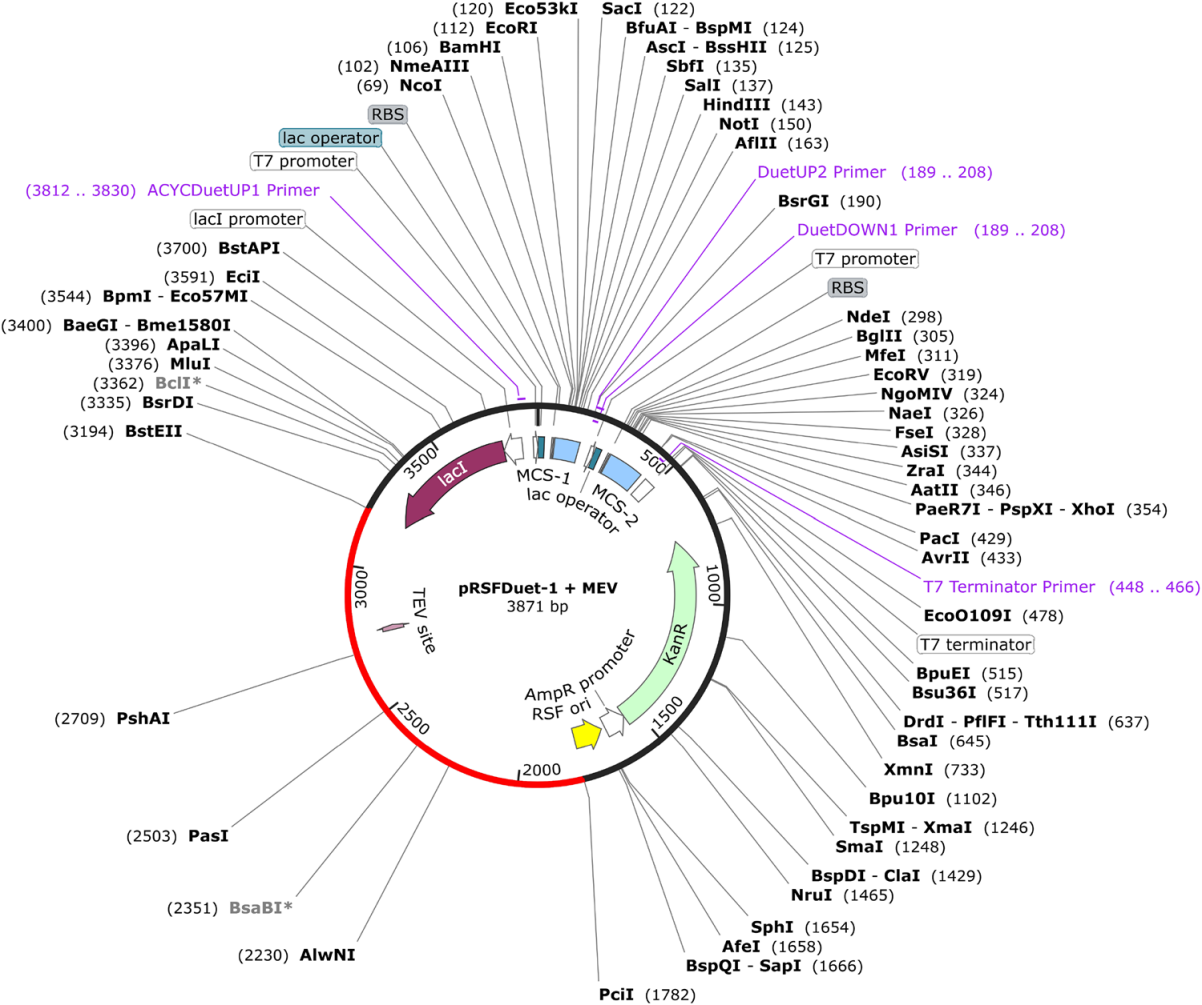
Building the finalised vaccine in pRSFDuet-1 vector in silico. The JCat server’s nucleotide sequence, which includes the codon sequence for the vaccine, is displayed in red. The pRSFDuet-1 expression vector is displayed in black. Software called SnapGene was used to make this figure.

Using the Java Codon Adaptation Tool, we assessed the expression efficiency and the codon utilisation of the vaccine design for cloning in *E. coli* strain K12. A total of 1412 nucleotides make up the optimised codon sequence. It has a Codon Adaptation Index (CAI) of 1.0, which is within (0.8–1.0), and an average GC content of 52.46%, which is within the ideal value (30–70%), both of which indicate a high possibility that the final vaccine would be adequately produced in the *E. coli* host. Using the SnapGene tool, we introduced the codon sequences into the pRSFDuet-1 vector see Fig. 8. It is placed between PciI (1782) and BstEII (3I94) locations in the vector. The final vaccine’s codon sequence is in red, and the pRSFDuet-1 expression vector is in black.

### Simulation of agarose gel electrophoresis and polymerase chain reaction

**Figure 9:**
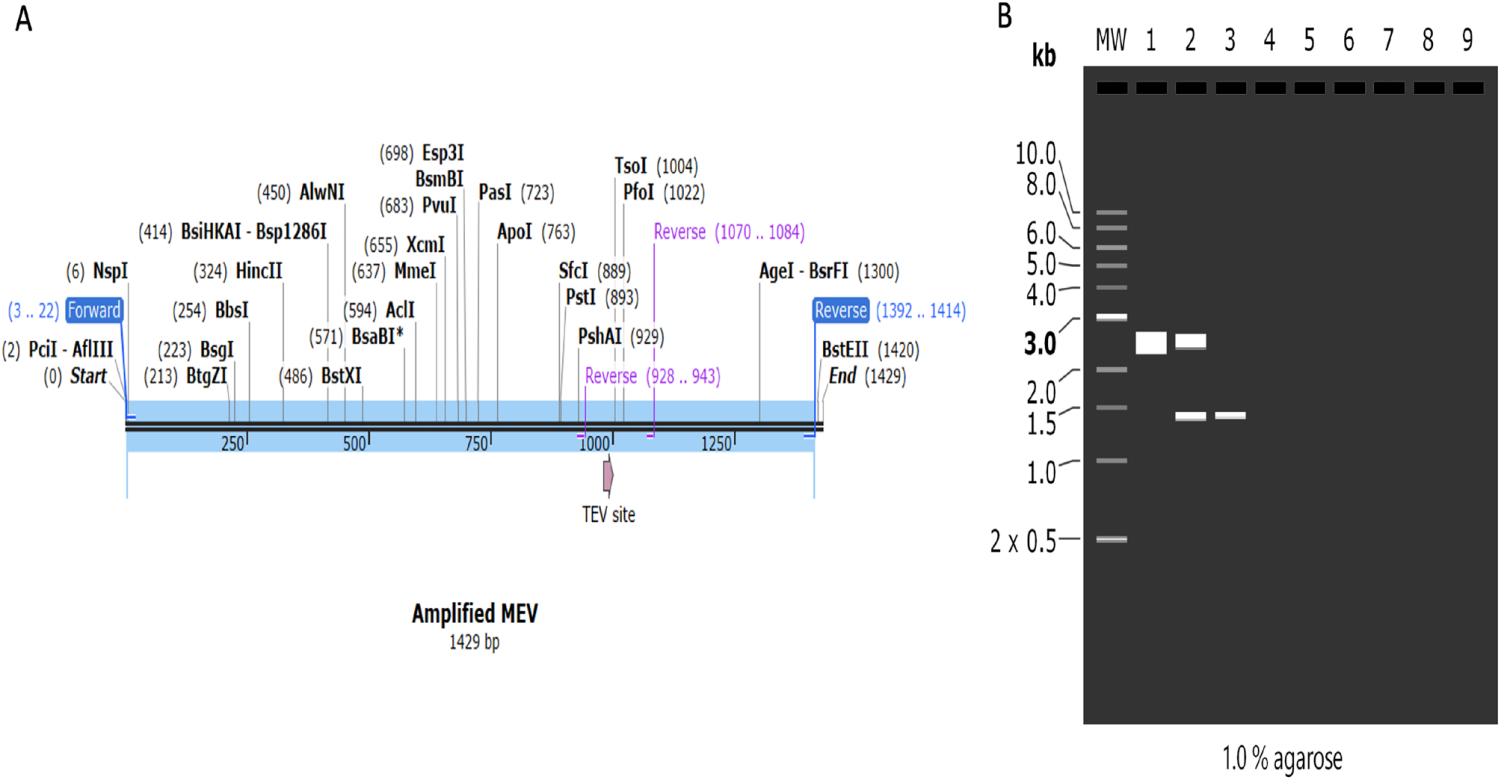
(**A**) The MEV following amplification (**B**) Simulation studies using agarose gel electrophoresis. The cloned pRSFDuet-1 plasmid + MEV (3871) lane 1, digestion of cloned pRSFDuet-1 plasmid + MEV with PciI, BstEII (2459 bp) and (1412 bp) lane 2, PCR amplified MEV ((1412 bp) is represented by lane 3.

A gel electrophoresis simulation was carried out to confirm whether the multi-epitope vaccine will be purified. The forward primer (5′-CATGTCTGGTTCTGACTTCG-3′) has a length of 20, a Tm value of 55 °C, and a GC content of 50%. The reverse primer (5′-CCGGACCCGGACCTTTACGGAAC-3′) has a length of 23, a Tm value of 65^0^C, and a GC content of 65%. These primers were created based on the parameters above. SnapGene was used to amplify the MEV’s target gene. We used a concentration of 1% for the simulated agarose electrophoresis, and we chose TBE based on its superior capacity to stabilise the target gene, vector, and recombinant plasmid in a buffer solution. Ultimately, the quantity of DNA matched earlier estimates. In the end, the amount of DNA matched with previous estimates. The amplified MEV sequence was 1412, and the cloned pRSFDuet-1 plasmid with MEVsequence was 3871 bp. After digestion of pRSFDuet-1 plasmid with PciI and BstEII enzymes, 2459 bp and 1412 bp sequences were obtained (Fig. 9).

### Simulation of immune response to vaccine

**Figure 10:**
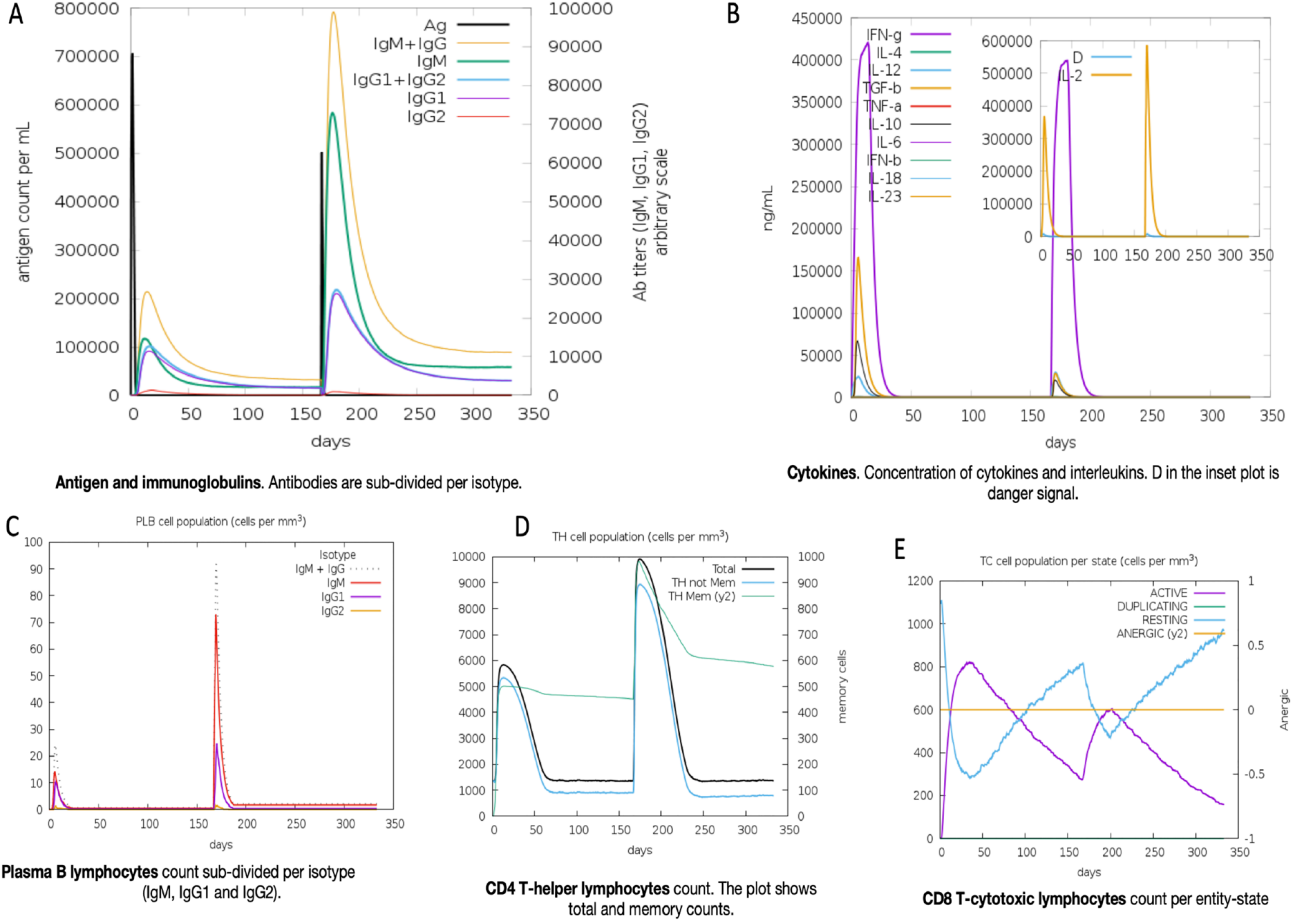
Utilising C-ImmSim to predict the immune simulation response to the vaccination design. C-ImmSim software conducted an in silico immune simulation research in response to delivering two doses of the vaccine design spaced five months apart.

Through the C-ImmSim server, our vaccine design significantly simulated the mammalian immune response (Fig. 10 A-E). The results also showed that our multi-epitopic vaccine produced a healthy number of B cells that secrete antibodies, with the IgM and IgG (Fig. 10A) subclasses being the most noticeable. The profile of cytokines (Fig. 10B) generated following the injections was one noteworthy finding. A dramatic surge of pro-inflammatory IFN-g and IL-2 after repeated vaccination was seen. Also, some anti-inflammatory cytokines, such as TGF-b and IL-6, showed peaks. Plasma cell (Fig. 10C) and T-cell (Fig. 10E) production were reasonably large, and memory cells (Fig. 10D) remained viable for several months. Therefore, our findings show that immune reactions gradually increased after a booster vaccination dose and lasted for several months.

## Conclusion

This study suggested a deep learning framework built on a biological optimisation algorithm for choosing epitopes that may be used in vaccine development. The DL-based algorithms have demonstrated exceptional performance for the prediction job and may thus be used to quickly and cheaply choose the appropriate epitopes to develop a SARS-CoV-2 vaccine. A multi-epitope vaccine against COVID-19 was created using the epitopes predicted by the model. The finalised vaccine’s toxicity, potential allergic reactions, and other physiochemical characteristics were examined and determined to be safe. Additionally, it has high antigenicity, which is crucial for producing a robust immunological response. Immune simulation findings demonstrated that our multi-epitope vaccination effectively generated sufficient B cells that release antibodies and T-cells.

Deep models used in vaccine development are considered black boxes as they extract predictions about peptide’s epitope status from the last layer without explanation. The SHAP technique explains these predictions, generating Shapley values for each feature to understand better its role in the neural network’s judgment and vaccine safety. Nevertheless, this study has some limitations. The interactions between the receptor and MEV were carried out using the TLR4 receptor, and other TLR receptors were used to confirm the probable interactions with the MEV. Although the tertiary structure, refinement, docking, and other physiochemical properties of the MEV have shown its quality and stability, the stability can be further confirmed using immune dynamic simulations. Finally, in vitro and in vivo experiments are needed to validate the efficacy of this multi-epitope vaccination. Further work will be carried out using other optimisation algorithms with deep explainable models that will provide more explanations regarding the prediction of epitopes for designing a vaccine against COVID-19.
